# Wastewater sequencing reveals early cryptic SARS-CoV-2 variant transmission

**DOI:** 10.1038/s41586-022-05049-6

**Published:** 2022-07-07

**Authors:** Smruthi Karthikeyan, Joshua I. Levy, Peter De Hoff, Greg Humphrey, Amanda Birmingham, Kristen Jepsen, Sawyer Farmer, Helena M. Tubb, Tommy Valles, Caitlin E. Tribelhorn, Rebecca Tsai, Stefan Aigner, Shashank Sathe, Niema Moshiri, Benjamin Henson, Adam M. Mark, Abbas Hakim, Nathan A. Baer, Tom Barber, Pedro Belda-Ferre, Marisol Chacón, Willi Cheung, Evelyn S. Cresini, Emily R. Eisner, Alma L. Lastrella, Elijah S. Lawrence, Clarisse A. Marotz, Toan T. Ngo, Tyler Ostrander, Ashley Plascencia, Rodolfo A. Salido, Phoebe Seaver, Elizabeth W. Smoot, Daniel McDonald, Robert M. Neuhard, Angela L. Scioscia, Alysson M. Satterlund, Elizabeth H. Simmons, Dismas B. Abelman, David Brenner, Judith C. Bruner, Anne Buckley, Michael Ellison, Jeffrey Gattas, Steven L. Gonias, Matt Hale, Faith Hawkins, Lydia Ikeda, Hemlata Jhaveri, Ted Johnson, Vince Kellen, Brendan Kremer, Gary Matthews, Ronald W. McLawhon, Pierre Ouillet, Daniel Park, Allorah Pradenas, Sharon Reed, Lindsay Riggs, Alison Sanders, Bradley Sollenberger, Angela Song, Benjamin White, Terri Winbush, Christine M. Aceves, Catelyn Anderson, Karthik Gangavarapu, Emory Hufbauer, Ezra Kurzban, Justin Lee, Nathaniel L. Matteson, Edyth Parker, Sarah A. Perkins, Karthik S. Ramesh, Refugio Robles-Sikisaka, Madison A. Schwab, Emily Spencer, Shirlee Wohl, Laura Nicholson, Ian H. McHardy, David P. Dimmock, Charlotte A. Hobbs, Omid Bakhtar, Aaron Harding, Art Mendoza, Alexandre Bolze, David Becker, Elizabeth T. Cirulli, Magnus Isaksson, Kelly M. Schiabor Barrett, Nicole L. Washington, John D. Malone, Ashleigh Murphy Schafer, Nikos Gurfield, Sarah Stous, Rebecca Fielding-Miller, Richard S. Garfein, Tommi Gaines, Cheryl Anderson, Natasha K. Martin, Robert Schooley, Brett Austin, Duncan R. MacCannell, Stephen F. Kingsmore, William Lee, Seema Shah, Eric McDonald, Alexander T. Yu, Mark Zeller, Kathleen M. Fisch, Christopher Longhurst, Patty Maysent, David Pride, Pradeep K. Khosla, Louise C. Laurent, Gene W. Yeo, Kristian G. Andersen, Rob Knight

**Affiliations:** 1 Department of Pediatrics, University of California San Diego, La Jolla, CA USA; 2 Department of Immunology and Microbiology, The Scripps Research Institute, La Jolla, CA USA; 3 Expedited COVID Identification Environment (EXCITE) Laboratory, Department of Pediatrics, University of California San Diego, La Jolla, CA USA; 4 Department of Obstetrics, Gynecology, and Reproductive Sciences, University of California San Diego, La Jolla, CA USA; 5 COVID-19 Detection, Investigation, Surveillance, Clinical, and Outbreak Response, California Department of Public Health, Richmond, CA USA; 6 Center for Computational Biology and Bioinformatics, University of California San Diego, La Jolla, CA USA; 7 Institute for Genomic Medicine, University of California San Diego, La Jolla, CA USA; 8 Department of Computer Science and Engineering, University of California San Diego, La Jolla, CA USA; 9 Operational Strategic Initiatives, University of California San Diego, La Jolla, CA USA; 10 Return to Learn, University of California San Diego, La Jolla, CA USA; 11 Student Health and Well-Being, University of California San Diego, La Jolla, CA USA; 12 Student Affairs, University of California San Diego, La Jolla, CA USA; 13 Academic Affairs, University of California San Diego, La Jolla, CA USA; 14 Department of Pathology, University of California San Diego, La Jolla, CA USA; 15 Scripps Health, San Diego, La Jolla, CA USA; 16 Rady Children’s Institute for Genomic Medicine, San Diego, CA USA; 17 Sharp Healthcare, San Diego, CA USA; 18 Helix, San Mateo, CA USA; 19 County of San Diego Health and Human Services Agency, San Diego, CA USA; 20 Herbert Wertheim School of Public Health and Human Longevity Science, University of California San Diego, La Jolla, CA USA; 21 Division of Infectious Disease and Global Public Health, University of California San Diego, La Jolla, CA USA; 22 Office of Advanced Molecular Detection, Centers for Disease Control and Prevention, Atlanta, GA USA; 23 Department of Biomedical Informatics, University of California San Diego, La Jolla, CA USA; 24 Office of the UC San Diego Health CEO, University of California San Diego, La Jolla, CA USA; 25 Department of Medicine, University of California San Diego, La Jolla, CA USA; 26 Sanford Consortium of Regenerative Medicine, University of California San Diego, La Jolla, CA USA; 27 Department of Cellular and Molecular Medicine, University of California San Diego, La Jolla, CA USA; 28 Department of Bioengineering, University of California San Diego, La Jolla, CA USA

**Keywords:** Environmental biotechnology, Genome informatics, SARS-CoV-2, Viral infection, Phylogenetics

## Abstract

As SARS-CoV-2 continues to spread and evolve, detecting emerging variants early is critical for public health interventions. Inferring lineage prevalence by clinical testing is infeasible at scale, especially in areas with limited resources, participation, or testing and/or sequencing capacity, which can also introduce biases^1–3^. SARS-CoV-2 RNA concentration in wastewater successfully tracks regional infection dynamics and provides less biased abundance estimates than clinical testing^4,5^. Tracking virus genomic sequences in wastewater would improve community prevalence estimates and detect emerging variants. However, two factors limit wastewater-based genomic surveillance: low-quality sequence data and inability to estimate relative lineage abundance in mixed samples. Here we resolve these critical issues to perform a high-resolution, 295-day wastewater and clinical sequencing effort, in the controlled environment of a large university campus and the broader context of the surrounding county. We developed and deployed improved virus concentration protocols and deconvolution software that fully resolve multiple virus strains from wastewater. We detected emerging variants of concern up to 14 days earlier in wastewater samples, and identified multiple instances of virus spread not captured by clinical genomic surveillance. Our study provides a scalable solution for wastewater genomic surveillance that allows early detection of SARS-CoV-2 variants and identification of cryptic transmission.

## Abstract (web summary)

Emerging SARS-CoV-2 variants of concern were detected early and multiple cases of virus spread not captured by clinical genomic surveillance were identified using high-resolution wastewater and clinical sequencing.

## Main

SARS-CoV-2 continues to evolve, producing diverse new lineages^6^. Emerging variants of concern (VOCs) and variants of interest (VOIs) demonstrate increased transmissibility, disease severity and/or immune escape^7^. Timely and accurate quantification of local prevalence of SARS-CoV-2 variants is thus essential for effective public health measures. However, existing strategies for variant detection based on virus genome sequencing of biospecimens obtained from clinical testing (‘clinical genomic surveillance’) are expensive, inefficient and have sampling bias because of systemic healthcare disparities, particularly in poor and underserved communities^1–3^.

By contrast, PCR-based wastewater surveillance of SARS-CoV-2 RNA is not subject to clinical testing biases and can track temporal changes in overall SARS-CoV-2 prevalence in a region^4,5,8^, but cannot identify epidemiological transmission links or monitor virus lineage prevalence, which require genome sequence information. Virus genome sequencing from wastewater (‘wastewater genomic surveillance’) has the potential to cost-effectively capture community virus spread^9,10^, acting as a surrogate to clinical surveillance in elucidating lineage geospatial distributions and track emerging SARS-CoV-2 variants (including new variants for which targeted assays do not yet exist), and provide genome sequence data needed for transmission network analysis and interpretation^11^.

However, wastewater genomic surveillance is technically challenging^10^. Low viral loads, heavily fragmented RNA and PCR inhibitors in complex environmental samples lead to poor sequencing coverage^12,13^. Obtaining high-quality sequences from samples with low viral load and elevated levels of PCR inhibitors remains an outstanding technical challenge in implementation of wastewater genomic surveillance at scale. In addition, tools for SARS-CoV-2 lineage classification, such as pangolin^14^ and UShER^15^, were designed for clinical samples containing a single dominant variant, and cannot estimate relative abundances of multiple SARS-CoV-2 lineages in samples with virus mixtures such as wastewater.

Here we report a high-resolution approach to study community virus transmission using wastewater genomic surveillance, leveraging several technical advances in wastewater virus concentration and nucleic acid sequencing, and a computational tool for resolving multiple SARS-CoV-2 lineages in short-read sequence data from a mixed sample (lineage deconvolution). We obtained near 95% genome coverage even for samples with low viral load, compared with 40% or below from previous studies^11–13^, a key advance that allowed us to build a robust pipeline to monitor virus lineage prevalence in community wastewater.

Because places of communal living, such as university campuses, are considered key sites for virus spread and represent well-controlled and relatively isolated environments, they are ideal for comparing the relative utility of clinical and wastewater genomic surveillance^16^. Accordingly, we conducted a high-resolution, longitudinal wastewater genomic surveillance effort at the University of California San Diego (UCSD) campus, in parallel with clinical genomic surveillance from nasal swabs in the local community, from November 2020 to September 2021: 10 months that effectively capture the surges in the region caused by the three main VOCs (as determined by the US Centers of Disease Control and Prevention (CDC)) in the United States: Epsilon, Alpha and Delta^6^. In more recent San Diego-wide data collected from September 2021 to February 2022, we studied ongoing transmission of the Delta variant and the rapid spread of the Omicron variant and its sublineages.

Our wastewater genomic surveillance approach identified VOCs up to 2 weeks before detection through clinical genomic surveillance, even though a large proportion of clinical SARS-CoV-2 samples is sequenced in San Diego relative to other cities in the United States. In addition to providing a detailed history of community virus spread, wastewater genomic surveillance also identified multiple instances of cryptic community transmission not observed through clinical genomic surveillance. Matching wastewater and clinical genome sequences provided epidemiological information identifying specific transmission events. Our results demonstrate the viability of wastewater genomic surveillance at scale, enabling early detection and tracking of virus lineages and guiding clinical genomic surveillance efforts. This work informed public health guidance and interventions on the UCSD campus as well as San Diego county in real time, and our data and analyses were disseminated to both public health officials as well as the general public via custom dashboards (see Data availability for links).

To directly compare wastewater genomic surveillance to clinical surveillance, we conducted a large-scale SARS-CoV-2 genome sequencing study from wastewater samples collected daily from 131 wastewater samplers covering 360 campus buildings, in many cases reaching single-building-level resolution. To identify epidemiological transmission links and monitor lineages in the population, we sequenced all clinical and wastewater samples positive for SARS-CoV-2 from campus using a miniaturized tiled-amplicon sequencing approach. During this period, we collected and analysed 21,419 wastewater samples: 19,944 wastewater samples from the UCSD campus, and, for comparison, 1,475 wastewater samples from the greater San Diego area, including the Point Loma wastewater treatment plant (the primary wastewater treatment plant for the county, with a catchment size of 2.3 million people) and 17 public schools spanning four San Diego school districts^17^. We compared sequencing of 600 campus wastewater samples to 759 genomes obtained from campus clinical swabs (46.2% of all positive tests on campus), all processed by the CALM and EXCITE CLIA laboratories at the UCSD. In addition, we compared 31,149 genomes obtained from clinical genomic surveillance of the greater San Diego community with sequencing of 837 wastewater samples collected from San Diego county (including those from the UCSD campus) during the same period.

## Uncovering microscale community spread

**Fig. 1:**
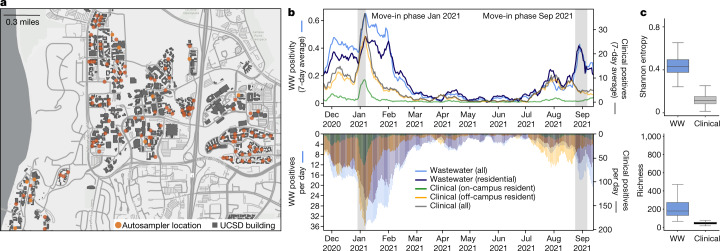
Campus sampling locations and SARS-CoV-2 testing statistics. **a**, Geospatial distribution of the 131 actively deployed wastewater autosamplers and the corresponding 360 university buildings on the campus sewer network. Building-specific data have been de-identified in accordance with university reporting policies. **b**, Campus wastewater (WW) and diagnostic testing statistics over the 295-day sampling period (positivity is the fraction of WW samplers with a positive qPCR signal). **c**, Virus diversity in wastewater and clinical samples; boxplots of Shannon entropy (top) and richness (bottom) for each sample type (*n* = 153 WW—a subset chosen to maximize sample independence; see Methods—and *n* = 5,888 clinical). Box edges specify the first and third quartiles, the solid line indicates the median, and the whiskers delimit the maximum and minimum values. Map in **a** is the intellectual property of Esri and its licensors and are used herein under license. Copyright © 2022 Esri and its licensors. All rights reserved.

**Extended Data Fig. 1:**
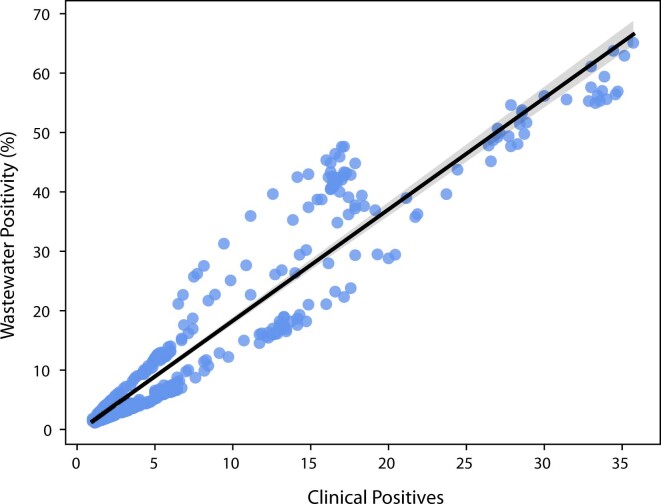
Relationship of daily UCSD campus wastewater sampler positivity and campus clinical positives. Black line indicates the linear regression fit (slope = 1.88 %/clinical positive, intercept = −0.45%) to the data (n = 321), with bootstrap 95% confidence interval (resampled 1000 times with replacement) shown in gray (median slope = 1.88%/clinical positive, intercept = −0.47%).

We implemented a geographical information system (GIS)-enabled building-level wastewater surveillance system to cover 360 buildings on the UCSD campus (Fig. 1a). During the period of daily wastewater sampling, approximately 10,000 students lived on campus and 25,000 individuals were on campus on a daily basis. We found that wastewater test positivity correlated strongly with the number of clinical positives (Fig. 1b and Extended Data Fig. 1), showing that wastewater effectively captures the community infection dynamics based on total viral load. This is also consistent with our past studies that showed that SARS-CoV-2 RNA can be detected approximately 85% of the time downstream from buildings containing individuals known to be infected^9^.

**Extended Data Fig. 2:**
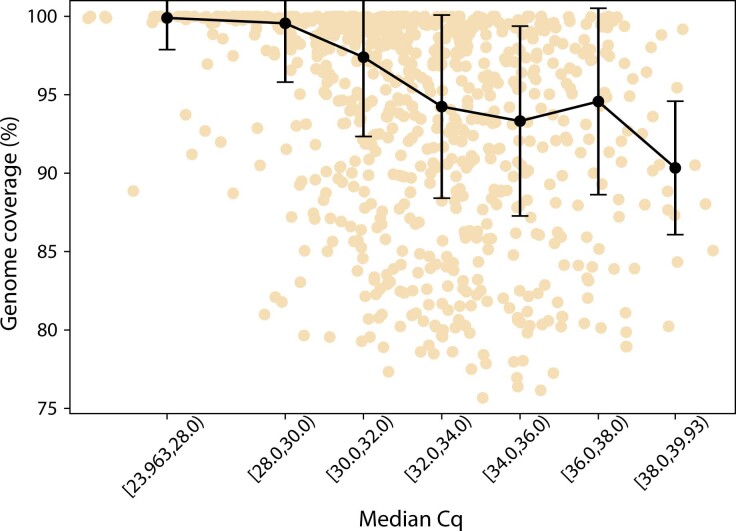
Relationship between genome coverage and cycle quantification values. 10x genome coverage (fraction of sites with 10 reads or greater) remains high, even for Cq values of nearly 38 (n = 786). Points indicate median value in each bin, while error bars indicate the median absolute deviation.

Unlike quantitative PCR (qPCR)-based mutant surveillance, genomic surveillance using full-length virus genomes can detect which strains of SARS-CoV-2 are circulating in the population, and can identify potential transmission links between infected individuals^18,19^. Although targeted qPCR mutant panels have the ability to detect specific lineages in wastewater, they only target a small set of mutations that must be known beforehand and require development and validation time before implementation. To test the utility of wastewater genomic surveillance for studying virus spread in the community, we obtained near-complete virus genomes for wastewater samples with cycle quantification values as high as 38 (median genome coverage of 96.49% (range 75.67–100.00%); Extended Data Fig. 2). However, using two common metrics of virus diversity—Shannon entropy (a measure of the uncertainty associated with randomly sampling an allele) and richness (the number of single-nucleotide variant (SNV) sites)^20^—we found that the genetic diversity of SARS-CoV-2 is significantly greater in wastewater samples than clinical samples (Fig. 1c; Mann–Whitney *U*-test, *P* < 0.001 for each, with effect size *r* = 0.99, 0.97 for Shannon entropy and richness, respectively). This suggests that multiple virus lineages, probably shed from different infected individuals, are often present in wastewater samples, whereas clinicalsamples generally contain a single virus lineage shed from one individual.

## Sample deconvolution robustly recovers the abundance of SARS-CoV-2 lineages in mixed samples

Wastewater systems aggregate stool, urine and other biological waste products carrying viruses from multiple infected individuals in the community in a single location, allowing for sampling of virus mixtures that are representative of local lineage prevalence. However, existing methods for determining virus lineage from sequencing are intended for non-mixed clinical samples and can only be used to identify a single (dominant) lineage per sample.

**Fig. 2:**
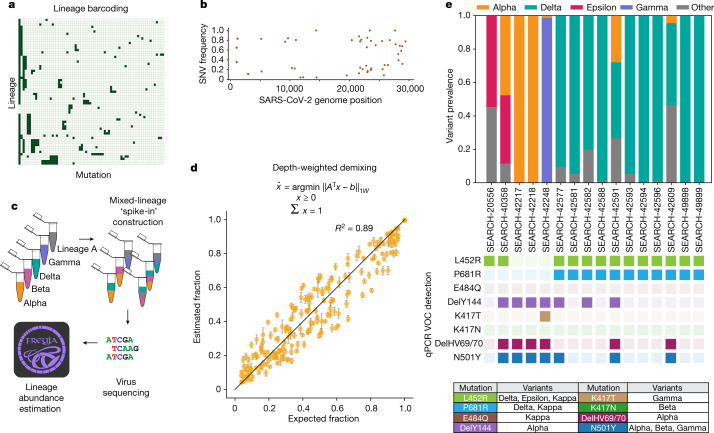
Sample deconvolution robustly recovers relative virus abundance. **a**, Subset of lineage defining mutation ‘barcode’ matrix. Each row represents one lineage (out of more than 1,000 lineages included in the UShER global phylogenetic tree), and individual nucleotide mutations are represented as columns. **b**, Single-nucleotide variant (SNV) frequencies obtained from iVar used for recovering relative abundance of each lineage. **c**, Schematic of the spike-in validation experiment. **d**, Depth-weighted demixing estimates of the virus abundance versus expected or known abundance. Details on lineage-specific predictions are provided in Extended Data Fig. 3. Error bars indicate s.d. of estimates across mixture replicates. **e**, Comparison of wastewater sample deconvolution with VOC qPCR panel, with lookup table (bottom) showing amino acid mutations corresponding to each variant.

To fully capture the virus diversity in community biospecimens, we developed Freyja, a tool to estimate the relative abundance of virus lineages in a mixed sample. Freyja uses a ‘barcode’ library of lineage-defining mutations to represent each SARS-CoV-2 lineage in the global phylogeny^21^ (Fig. 2a). To encode each sample, Freyja stores the SNV frequencies (the proportion of reads at a site that contain the SNV) for each of the lineage-defining mutations (Fig. 2b, top). As SNV frequencies at positions with greater sequencing depth more accurately estimate the true mutation frequency, Freyja recovers relative lineage abundance by solving a depth-weighted least absolute deviation regression problem, a mixed sample analogue of minimizing the edit distance between sequences and a reference (Fig. 2b, bottom). To ensure results are meaningful, Freyja constrains the solution space such that each lineage abundance value is non-negative and overall lineage abundance sums to one. Freyja performs site-specific weighting to account for non-constant variance in measured SNV frequency across sites, enabling prioritization of information at each site as a function of sequencing depth. Read depths were log-transformed, providing robustness to common attributes of real sequencing data such as heavily skewed read depth across amplicons.

**Extended Data Fig. 3:**
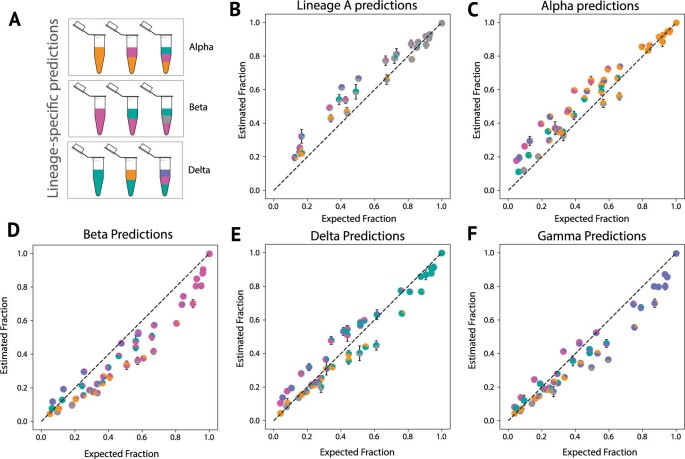
Lineage-specific prediction of variant abundance in spike-in validation samples. A. Schematic of “spike-in” sample design. B-F. Lineage specific prediction. Proportions of each lineage in the sample are shown as a pie chart marker (Grey = Lineage A, Orange = Alpha, Pink = Beta, Turquoise = Delta, and Purple = Gamma) with error bars indicating the standard deviation from the mean, across four replicates (n = 380, four samples per mixture type).

To validate Freyja, we sequenced ‘spike-in’ synthetic mixtures from five key SARS-CoV-2 lineages (lineage A, Beta, Delta, Epsilon and Gamma) at proportions ranging from 5% to 100% in each sample, with between one and five different lineages per mixture (Fig. 2c and Extended Data Table 1). We found that Freyja robustly recovered the expected lineage abundances for all mixtures, even for lineages at 5% abundance (Fig. 2d and see Extended Data Fig. 3 for lineage-specific predictions). To further validate Freyja, we used wastewater samples from the UCSD isolation dorms as well as Point Loma wastewater treatment plant, collection sites likely to contain mixed-lineage samples, to compare Freyja-detected lineages with qPCR testing for eight mutations associated with different VOCs (N501Y, DelHV69/70, DelY144, K417N, K417T, E484Q, P681R and L452R; Fig. 2e). We found that Freyja consistently identified the same lineages as qPCR testing, but, as expected, also identified additional lineages with SNVs not included in our qPCR panel that were known to be circulating in San Diego at the time of collection. Combined, these results show that Freyja robustly estimates viral lineage abundance from samples containing a mixture of lineages, including synthetic virus mixtures and field wastewater collections.

**Extended Data Fig. 4:**
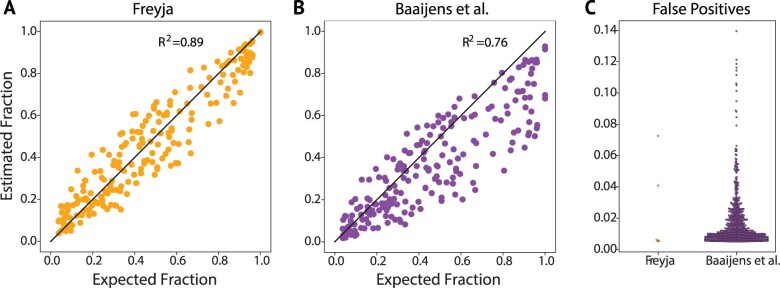
Freyja more accurately estimates virus abundance, with fewer false positives. A-B. Estimated vs expected fraction of each lineage in the mixture (n = 95, one sample per mixture type). The Kallisto-based approach from Baaijens et. al shows a wider range of estimates for each known mix fraction, and generally underestimates the fraction. C. False positives with abundance greater than 0.5%.

To compare Freyja with other wastewater analysis pipelines, we tested the performance of other wastewater deconvolution methods including a method from Baaijens et al.^12^, cojac^22^ and Lineage deComposition for SARS-CoV-2 (LCS)^23^ using the spike-in mixtures (Extended Data Fig. 4). We found that Freyja greatly outperformedf other methods in terms of accuracy, false-positive rate and computational efficiency. The method from Baaijens et al.^12^ required more than ten times more computation time per sample relative to Freyja (approximately 13.2 min versus approximately 1.1 min per sample, respectively). Although cojac was fast, the small amplicon length used for the spike-in mixtures resulted in cojac failing to identify most of the variants entirely, whereas LCS failed to return estimates within 2 days.

## Detection of community transmission in wastewater

**Fig. 3:**
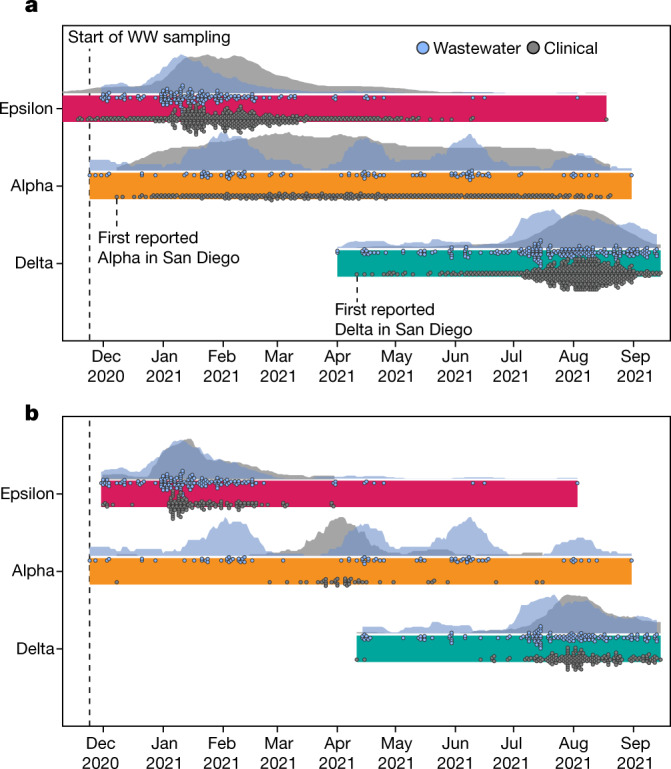
Freyja recovers early and cryptic transmission of SARS-CoV-2 variants of concern. **a**, Timeline and normalized epidemiological curves for VOC detection in both wastewater and clinical sequences from San Diego County (includes wastewater samples collected from Point Loma wastewater treatment plant, UCSD, as well as public schools in the San Diego districts) for the three major VOCs in circulation during the sampling period (*n* = 475 wastewater, *n* = 22,504 clinical). Both Alpha and Delta variants are detected first in wastewater before clinical samples. Markers for clinical detections correspond to the ceiling of the daily detection count divided by 30 (for example, 1–30 samples = one marker, 31–60 = two markers), whereas wastewater markers correspond to a single detection. **b**, Timeline and epidemiological curves for VOC detection in the campus samples (*n* = 364 wastewater, *n* = 333 clinical). Markers correspond to a single detection event for both clinical and wastewater surveillance. All wastewater detections correspond to an estimated VOC prevalence of at least 10%.

**Extended Data Fig. 5:**
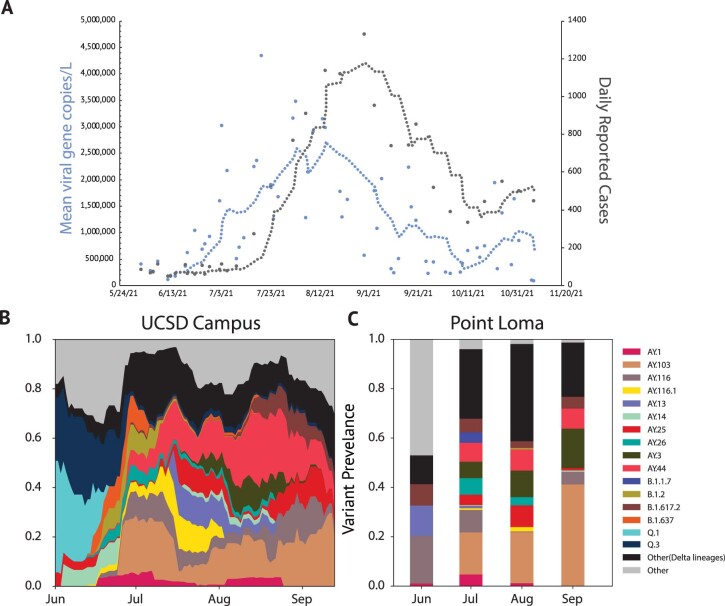
The rise of the Delta variant during Summer 2021. A. Mean SARS-CoV-2 viral gene copies/L of raw sewage (blue) collected from the Point Loma Wastewater Treatment Plant and caseload (gray) reported by the county during the same period. SARS-CoV-2 concentrations were normalized by PMMoV (pepper mild mottle virus) concentration to adjust for load changes. B. Lineage distribution in UCSD campus wastewater. C. Monthly lineage averages for wastewater collected at Point Loma Wastewater Treatment Plant during the Delta surge (N = 5, 20, 25, 7).

**Extended Data Fig. 6:**
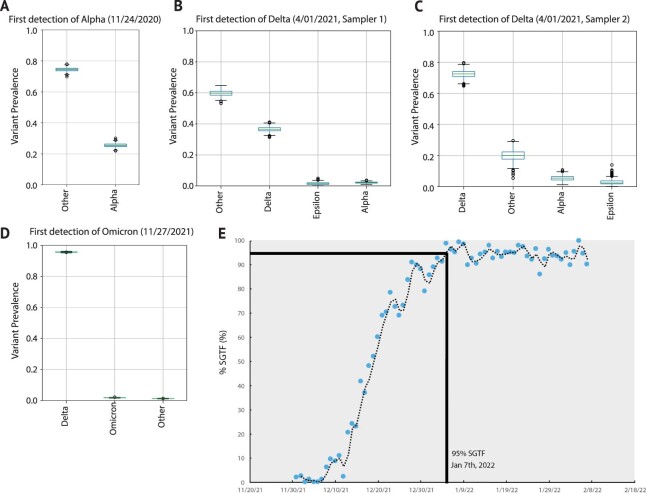
Quantification of deconvolution uncertainty in first detection of VOCs. A-D. Bootstrap distributions of Freyja abundance estimates obtained by resampling read data from each sample corresponding to the first detection of that VOC in San Diego 1000 times with replacement. In all boxplots, box edges specify the first and third quartiles, solid line indicates the median, and whiskers delimit the maximum and minimum values within 1.5 times the inter-quartile range (IQR) of box edges. Outliers are denoted with individual markers. Two samplers were found to contain Delta on the same day. First detections were also confirmed using a VOC qPCR panel, as shown in Fig. 2 and Extended Data Table 3. 95% Confidence intervals for variant prevalence for each first detection event: A. Alpha: (0.232, 0.278), B. Delta: (0.336, 0.397), C. Delta: (0.676, 0.772), D. Omicron: (0.017, 0.021). **E. Estimated proportion of Omicron sequences in clinical data**. Omicron estimates tracked via S-gene target failure, SGTF (characteristic of Omicron lineage BA.1 and its descendants) qPCR assays for clinical samples in San Diego between November 27th, 2021-February 7th, 2022. First detection of Omicron through clinical genomic sequencing in San Diego was December 8th. Dotted line shows a rolling average with a window size of seven days.

SARS-CoV-2 RNA concentrations in wastewater have been shown to be an early indicator of rising COVID-19 community incidence^9,24^ (Extended Data Fig. 5A), but whether wastewater can be used to detect emerging variants, including VOCs and VOIs, before their observation in clinical surveillance is unknown. To test whether wastewater can enable early detection of emerging lineages, we applied Freyja to our wastewater sequencing data and compared the collection date of VOC-positive samples from wastewater with the collection dates of samples from clinical genomic surveillance (Fig. 3a). With only 2.6% as many sequenced wastewater samples as sequenced clinical samples, we detected the Alpha and Delta VOC lineages in wastewater genomic surveillance up to 14 days before their first detection in genomic clinical surveillance (Epsilon was circulating at the start of wastewater collection, and thus could not be detected early). To further quantify our uncertainty in prevalence estimates, we used a fast bootstrapping approach (Extended Data Fig. 6) and found that the resampled distributions did not include zero abundance. As emerging VOC lineages may evade immune responses or lessen the effectiveness of public health interventions^18^, this early detection provides additional time to make necessary adjustments to existing countermeasures.

To test whether wastewater genomic surveillance can identify changes in the abundance of circulating lineages, we compared VOC detection rates in clinical and wastewater sequencing over time. We found that both wastewater and clinical genomic surveillance tracked changes in lineage abundance, but increases in lineage detection frequency were generally observed first in wastewater surveillance. For example, for the Epsilon variant, which was first detected in San Diego in September 2020, we observed increases in detection frequency in wastewater approximately 5 days before the corresponding increase in clinical genomic surveillance data (Fig. 3a; see Methods). We noticed varying periods of ongoing lineage detection across VOCs relative to clinical surveillance, possibly due to different virus shedding characteristics across lineages^25^. For Epsilon specifically, elevated sampling density on the UCSD campus relative to elsewhere in the county early on in the experiment may have biased San Diego-wide detection trends towards campus trends, particularly during the end of the wave. We also observed clear signatures of times with increased travel, as seen in the pulsing of Alpha detections in wastewater around the end of holidays and school breaks. During these periods as well as other times of mass student arrival, students were mandated to test immediately on arrival before they moved into their respective on-campus housing. In late March 2021 following the university break, mandated clinical testing identified the spread of the Alpha variant exclusively in off-campus residents (Fig. 1b), suggesting that campus mitigation protocols kept the Alpha outbreak from spreading on campus during this period.

To study the effectiveness of wastewater genomic surveillance at a smaller community scale, we restricted our analysis to samples from the UCSD campus. We found that wastewater genomic surveillance consistently identified the three major VOCs (Epsilon, Alpha and Delta) throughout their period of occurrence, despite detection gaps of 1 month or longer in clinical surveillance that included regular asymptomatic testing, longer than the expected signal due to extended virus shedding^26–28^ (Fig. 3b). During these gaps, positive samples were collected from multiple distinct locations, with most locations not repeated, suggesting that this continued detection in wastewater was not simply due to extended shedding. From mid-December to late March, the Alpha variant was detected more than once per week on average in wastewater but was not detected by clinical surveillance. Similarly, wastewater surveillance detected continued Delta transmission from mid-April to mid-June, but no cases were identified by clinical surveillance. This explains, in part, the long tails of wastewater positivity on campus relative to clinical surveillance on campus (Fig. 1b), in which we controlled for extended shedding by excluding samples from campus isolation dorms (see Methods for details). The high wastewater positivity level in February–March 2020 extends beyond the expected duration of extended shedding, indicating that cryptic transmission probably had a substantial role in the spread of the virus on campus during this period.

**Fig. 4:**
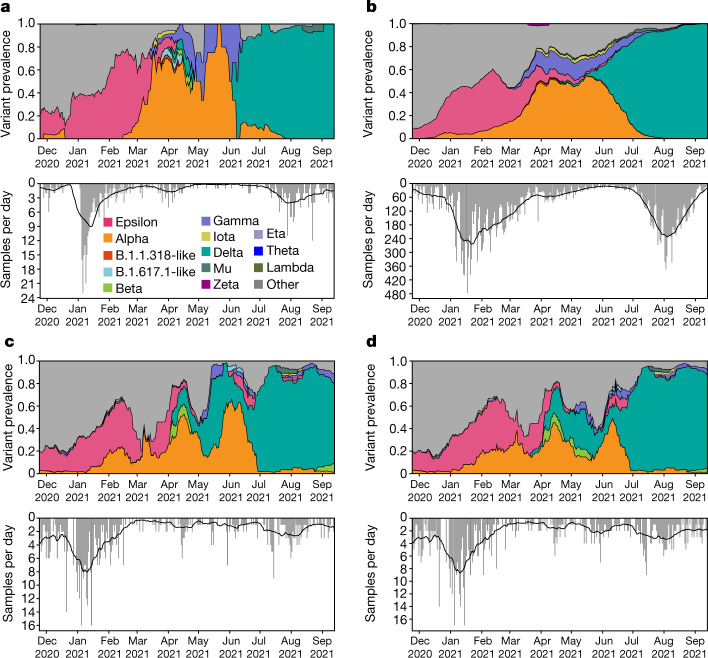
Deconvolution recovers a fine-grained estimate of virus population dynamics. **a**,**b**, Prevalence of SARS-CoV-2 variants in UCSD clinical surveillance (**a**) and variant prevalence in all clinical samples collected in San Diego County (**b**). **c**,**d**, Variant prevalence in wastewater at UCSD (**c**) and the greater San Diego County (**d**). Further analysis of Point Loma wastewater samples is shown in Extended Data Fig. 5. All curves show the rolling average, with a window of ±10 days. ‘Other’ contains all lineages not designated as VOCs. The bottom panels show the number of sequenced samples per day.

To study the effectiveness of wastewater surveillance in detecting and tracking other emerging variants, we aggregated all wastewater sequencing data to estimate the temporal profile of community lineage prevalence. We found that estimates of lineage abundance using wastewater enable early identification of other VOCs or VOIs, even for lineages that are rarely observed in clinical surveillance (Fig. 4). For example, we detected the Mu (B.1.621) variant via wastewater genomic surveillance on 27 July, nearly 4 weeks before its first detection through clinical genomic surveillance on campus on 23 August (Fig. 4a,c). However, despite persistent Mu detection in campus wastewater throughout July and early August, we did not detect the Mu variant in clinical or wastewater genomic surveillance on campus in September, suggesting that local community transmission did not continue.

To test whether Freyja continues to provide representative estimates of lineage prevalence for mixtures containing closely related lineages, we analysed the rise of the Delta variant (B.1.617.2) and its sublineages (AY.*) in San Diego, from June to September 2021 (Extended Data Fig. 5B,C). At both the UCSD campus and the Point Loma wastewater treatment plant, we identified the rapid emergence of B.1.617.2 and its sublineages (AY.*), along with low but persistent levels of the P.1 (Gamma) variant. The relative abundances of each of the variants were within twofold of prevalence estimates observed in clinical nasal swab data, suggesting that Freyja effectively identifies prevalence even for closely related lineages, at both the university and the county scale.

**Fig. 5:**
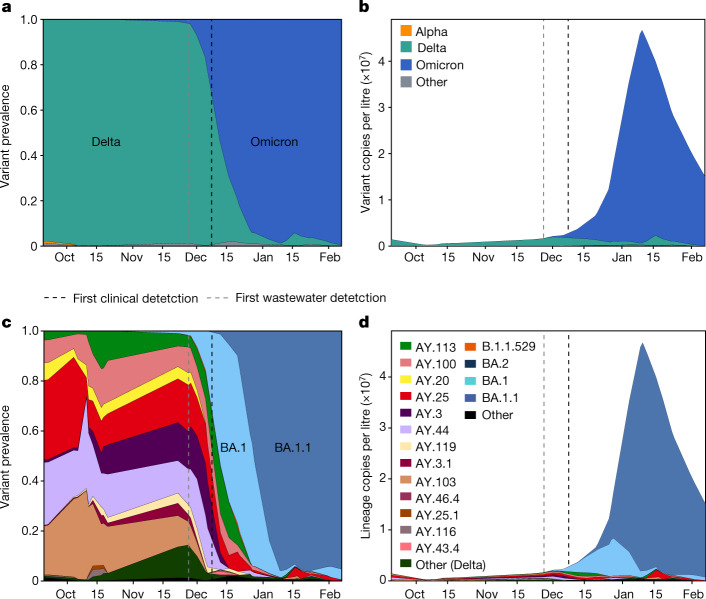
Community wastewater enables early Omicron detection and reveals lineage dynamics. **a**, Prevalence of SARS-CoV-2 VOCs in wastewater collected from the Point Loma wastewater treatment plant from late September 2021 to early February 2022. **b**, Estimated VOC concentrations; prevalence estimates were scaled by normalized viral load in wastewater. **c**,**d**, Lineage-specific estimates of prevalence (**c**) and concentration (**d**). All curves show an adaptive rolling average calculated using a local linear approximation (Savitzky–Golay filter) of virus copies per litre, with a window size of ±1 sampling date.

From more recent data from Point Loma wastewater treatment plant, we identified the Omicron variant (B.1.1.529 and descendants) at an abundance of near 1.7% on 27 November, more than 10 days before the first clinical detection in San Diego on 8 December (Fig. 5a,b). To confirm these findings, we applied our VOC qPCR panel to the same samples and consistently detected two mutations associated with the Omicron variant (DelHV69/70 and N501Y) in samples detected after 27 November, whereas neither was detected in samples from earlier in November (Extended Data Table 3; P681R was included to confirm the presence of Delta**)**.

To visualize the dynamics of competition between the Delta and Omicron variants, we analysed wastewater collected at Point Loma from late September through to early February. We found that upon introduction to the community, Omicron rapidly rose to dominance and reached roughly 95% prevalence by 26 December. During the same period, the estimates for 95% Omicron abundance in clinical samples tracked via S-gene target failures was 7 January, further suggesting that wastewater genomic surveillance is a leading indicator of lineage dynamics for emerging variants (Fig. 5a and Extended Data Fig. 6E). To understand the magnitude of lineage abundance, we scaled each sample by the measured virus RNA concentration of the sample (Fig. 5b). We observed that the absolute amount of circulating Delta variant remained largely constant upon the introduction of Omicron, even as it appeared to decrease to a small fraction of all viruses in the community.

To study the contribution of individual virus lineages to virus RNA concentration, we further analysed the growth dynamics of Delta and Omicron sublineages (Fig. 5c,d). We found that the many Delta lineages circulating in October and November were rapidly displaced by the BA.1 Omicron lineage, which was soon after displaced by the BA.1.1 lineage, suggesting a growth advantage over BA.1 and B.1.1.529. We did not observe significant levels of any other Omicron sublineages.

## Wastewater reveals history of campus infections

**Fig. 6:**
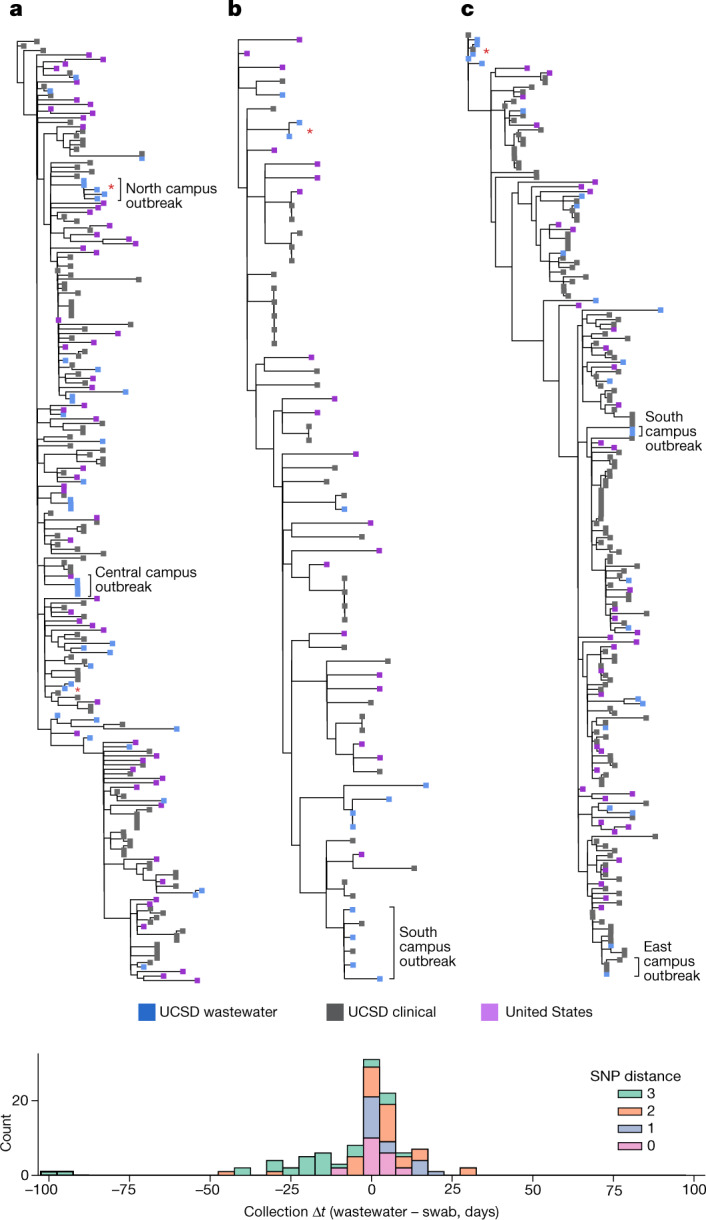
Wastewater identifies clinically known and unknown virus transmission. **a**–**c**, Maximum likelihood phylogenetic trees for each of the dominant VOCs (Epsilon (**a**), Alpha (**b**) and Delta (**c**)) using high-quality samples obtained at UCSD, as well as a representative set of sequences from the entire United States. Wastewater sequences from the same sampler that differ by one or fewer SNPs are denoted with a red asterisk. For all sequences, consensus bases were called at sites with more than 50% nucleotide frequency. Location information is provided for select outbreaks. **d**, Pairwise comparison of collection date for matching and near-matching wastewater and nasal swab samples obtained at UCSD. Positive values indicate earlier collection in nasal swabs and negative values indicate earlier detection in wastewater.

Phylogenetic analysis of virus genomes can be used to identify fine-scale spatial and temporal transmission networks, but it is not known whether wastewater can be used to further refine possible sites of transmission, elucidate transmission networks (‘who infected whom’) or identify specific infected individuals^19^. To investigate the scale, structure and timing of SARS-CoV-2 spread on campus, we reconstructed a maximum likelihood phylogenetic tree for each of the major VOCs using all high-quality consensus genomes (see Methods for details) obtained from the UCSD campus, as well as reference sequences for each lineage obtained elsewhere in the United States (Fig. 6a–c). In each tree, we identified many independent introductions, some of which led to extended transmission on campus. The resulting virus diversity among the VOCs present on campus enables ruling out of most transmission links and suggests that the spread of virus on campus comprised many separate, small outbreaks.

**Extended Data Fig. 7:**
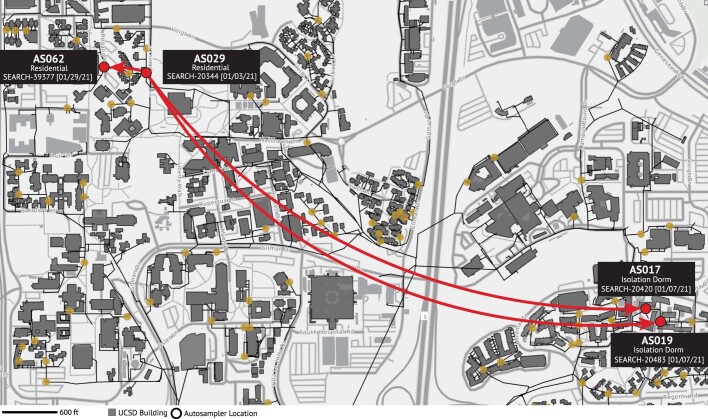
Temporal and spatial dynamics of an Epsilon outbreak at UCSD. After initial detection on January 3rd 2021, infected individuals were transferred to isolation housing where they continued to shed virus. At the end of January, a matching virus was detected in a residence nearby the original site of detection. All four samples have perfectly matching virus genomes. Maps are the intellectual property of Esri and its licensors and are used herein under license. Copyright © 2022 Esri and its licensors. All rights reserved.

To analyse the spatial structure of virus spread, we identified collection sites for wastewater sequences connected to transmission chains on campus, with building-specific resolution (Fig. 6a–c and see detailed example in Extended Data Fig. 7). We observed multiple small, linked outbreaks clustered in nearby buildings. Campus isolation protocol required students in congregate living to relocate to an isolation dorm, and linkages in the wastewater samples from buildings used for isolation reflected this co-location. We also found multiple instances of successive exactly matching sequences from wastewater collected from a single building, possibly due to continued viral shedding from the same infected individuals from extended shedding in stool^26–28^ or a transmission chain in the building leading to multiple infections by genetically identical viruses.

To study the temporal delay between clinical and wastewater lineage detection, we compared collection times of sequences from campus wastewater that match sequences from campus clinical surveillance (including non-VOC lineages). We found 20 exact sequence matches and 103 near-matches (SNP distance of three or less), but did not observe any overall bias towards earlier or later detection in wastewater (Fig. 6d), suggesting that, on average, wastewater and clinical genomic surveillance identify a similar timing of individual detection events. However, despite current technical difficulties with isolating haplotypes from diverse virus mixtures, more than half of the clinical–wastewater sequence pairs demonstrate earlier detection in wastewater or are from the same date. As detection is often delayed or missed by clinical surveillance, detections occur first in wastewater (despite a loss of sequences due to limited haplotype recovery), further suggesting that wastewater genomic surveillance can reveal the presence of specific genome sequences before clinical surveillance.

## Discussion

We show that improved virus concentration from wastewater, coupled with a method for resolving multiple lineages from mixed samples, captures community virus lineage prevalence and enables early detection of emerging variants, often before observation in clinical surveillance. By sequencing both clinical and wastewater samples from the UCSD campus, we detected VOCs persistently in wastewater even when their appearance in clinical samples is intermittent. However, we also found occasions when rarer lineages, such as B.1.1.318, were detected in clinical samples but not in wastewater. This is not unexpected on campus as many students living off-campus did not contribute to campus wastewater but were still clinically tested as part of testing mandates and policies. In the larger San Diego community context, this suggests that we may not be able to identify lineages circulating at low prevalence (less than 1%) using a single wastewater collection site. In addition, we note that clinical sequences identified from the community may not be observable in the contributing catchment, as precise geolocation of all clinical samples was not possible. Conversely, we also observed rare lineages in wastewater not seen in clinical samples from the campus or the community. As campus testing mandates are unable to capture all cases (for example, fully vaccinated individuals were not required to test and not all community samples were sequenced), rare lineages can be missed.

The considerable benefits of wastewater surveillance may stem from biases in clinical testing, including population testing availability and compliance, university quarantine policies and asymptomatic transmission, which may distort estimates of virus lineage prevalence from clinical samples. Wastewater offers less biased and more consistent viral lineage prevalence estimates, especially in areas with limited access and/or higher testing hesitancy rates, in which limited clinical surveillance can delay detection of emerging variants. As it requires considerably fewer samples, it is also more cost-effective than clinical testing and could serve as a long-term passive surveillance tool. This is particularly important for developing public health interventions in low-resource and underserved communities, where widespread clinical genomic surveillance for SARS-CoV-2 remains limited.

Wastewater is an information-dense resource for estimating the prevalence of specific viral lineages, providing a community-wide snapshot not only of overall infection dynamics but also of the rise and fall of specific VOCs. Our method, Freyja, deconvolutes these information-rich mixtures of virus lineages. For a large catchment area, such as the Point Loma wastewater treatment plant in San Diego, which covers over 2 million residents, even limited sampling may accurately estimate lineage prevalence in the population and provide an early warning indicator of the rise of new VOCs (as evidenced by the detection of Omicron at just over 1% abundance 11 days ahead of the first local clinical observation). In addition, wastewater genomic surveillance with building-level resolution provides a detailed description of the structure and dynamics of community virus transmission and can identify transmission links. It can be used to better direct public health interventions and can do so in real-time when combined with fast-turnaround sequencing technologies. This high-resolution approach is of particular utility in community gathering and transit sites, such as schools and airports, as well as sites with highly vulnerable individuals, such as nursing homes and hospitals, where spatially resolved monitoring for directing public health interventions is of great importance.

As SARS-CoV-2 continues to evolve, the risk of new VOCs remains high and there is a growing need to identify these viruses ahead of their proliferation in the community. Accordingly, development of technologies that are cost-effective, reduce biases and provide leading rather than trailing indicators of infection are essential to removing ‘blind spots’ in our understanding of local virus dynamics. Although technical issues have made wastewater sequencing difficult to perform at scale, our key advances in virus concentration and sample deconvolution provide evidence that this approach is now viable. Continued improvements to sequencing turnaround speeds, lineage barcoding and haplotype recovery from mixed samples will further accelerate efforts to achieve earlier identification of emerging variants and improve the precision and effectiveness of interventions.

## Methods

### Ethics declarations

The UCSD Institutional Review Board (IRB) provided human subject protection oversight of the data obtained by the EXCITE laboratory for the campus clinical samples (IRB approval nos. #210699 and #200477). All necessary patient and/or participant consent was obtained and the appropriate institutional forms have been archived, and any sample identifiers included were de-identified. The wastewater component of this project was discussed with our IRB, and was not deemed to be human subject research as it did not record personally identifiable information.

### Wastewater sampling

#### High-resolution spatial sampling at the campus level

One hundred and thirty-one wastewater autosamplers collecting 24-h time-weighted composites were deployed across manholes or sewer cleanouts of 360 campus buildings. GIS-informed analyses as well as agent-based network modelling of SARS-CoV-2 transmission on the UCSD campus enabled identification of most optimal locations for wastewater sampling. During the pilot phase (23 November to 29 December 2020), 68 samplers were prioritized to cover 239 residential buildings identified as the highest risk areas for large outbreaks on campus as a part of an observational study of wastewater monitoring in high-density buildings^29^. This was on the basis of preliminary dynamic modelling, which showed the largest potential outbreaks to occur within the largest residential buildings^9^. In addition to the observational study of wastewater monitoring in these high-density buildings, a cluster randomized study was also performed concurrently. This included a randomized modified version of a stepped wedge crossover design, in which there was random assignment of manholes for wastewater sampling. Clusters of manholes associated with residential buildings were randomized to receive wastewater monitors at one of two-time steps to evaluate the effect of wastewater monitoring on outbreak size in the associated buildings. During the same time period, all students in these residences were mandated to undergo weekly diagnostic testing, which was used to validate the utility of building-level wastewater monitoring. Furthermore, on-campus residences were initially focused due to the relatively static nature of the population, which enabled a more robust cross-validation of the sensitivity and efficacy of the wastewater surveillance. The coverage of wastewater surveillance was then increased to cover the rest of the campus buildings (including non-residential buildings on campus) from January 2021. Four of the deployed wastewater samplers covered the designated isolation and quarantine buildings on campus.

Wastewater composites were collected from the 131 samplers every day for the on-campus residence buildings and Monday through to Friday for the non-residential campus buildings. Wastewater samples (*n* = 19,944) were collected and analysed for the presence of SARS-CoV-2 RNA via qPCR with reverse transcription (RT–qPCR) between 23 November 2020 and 20 September 2021. During this time, 9,700 students lived in campus residences and 25,000 worked on campus on a daily basis. Between October 2020 to 1 January 2021, all on-campus residents were mandated to test on a biweekly (once every 2 weeks) basis and on a weekly basis from 2 January 2021 (start of the winter term). However, fully vaccinated individuals were not mandated to test on a regular basis. Campus protocols required students positive for SARS-CoV-2 living in congregate housing to relocate to designated isolation housing. Accordingly, our analysis of wastewater positivity (Fig. 1b) did not include isolation housing samplers, to control—as best as possible, as a small number of students in non-congregate housing spaces were allowed to isolate ‘in place’, for example—for possible repeat detection due to extended shedding from infected individuals. Automated, localized wastewater-triggered notifications were sent to the residents and/or employees of buildings associated with a positive wastewater signal, which further led to a surge in testing uptake rates by 2- to 40-fold in the associated buildings.

#### Wastewater sampling at the county level

Twenty-four-hour flow-weighted composites were collected three times a week from the main pump station for the Point Loma wastewater treatment plant, which is the primary treatment plant serving the greater San Diego county with a catchment size of approximately 2.3 million. Wastewater samples (*n* = 132) were collected between 24 February 2021 to 7 February 2022.

### Wastewater sample processing and viral genome sequencing

#### Sample processing

SARS-CoV-2 RNA was concentrated from 10 ml of raw sewage and processed as described elsewhere^4^. In brief, the viral RNA was concentrated using an automated affinity capture magnetic hydrogel particle (Ceres Nanosciences Inc.) based concentration method after which the nucleic acid was extracted and sample eluted in 50 µl of elution buffer. The extracted RNA was then screened for SARS-CoV-2 RNA via real-time RT–qPCR for three gene targets (N1, N2 and E-gene). PMMoV (pepper mild mottle virus) was also screened to adjust for changes in load. Positive wastewater samples were sequenced within 1–2 weeks of collection, comparable to the delay for clinical samples. To cross-validate the ability of the deconvolution tool in reliably resolving mixtures of strains in wastewater, the wastewater samples from the county as well as the samples from the isolation dorms on campus (where multiple infected individuals were isolating) were also run through a PCR panel targeting eight mutations associated with the strains designated as VOCs. The mutations screened for in wastewater using RT–qPCR included N501Y, DelHV69/70, DelY144, K417N, K417T, E484Q, P681R and L452R (CS3174B02, Promega Corp.).

#### Miniaturized wastewater SARS-CoV-2 amplicon sequencing

The Swift Normalase Amplicon Panels (SNAP) kit (PN: SN-5X296 (core) COVG1V2-96 (amplicon primers), Integrated DNA Technologies) was used on RNA from wastewater samples that were positive for SARS-CoV-2 RNA to prepare the multiplex next-generation sequencing (NGS) amplicon libraries and indexed using the SN91384 series of dual indexing oligos, yielding up to 1,536 index pairs per pool. A miniaturized version of the protocol was used with the following modifications: the Superscript IV VILO (Thermo Fisher) cDNA synthesis reaction was scaled down to approximately one-twelfth the normal reaction volume with 0.333 µl of enzyme mix and 1.333 µl of RNA being used. The multiplex amplicon amplification and Ampure XP bead purification steps were scaled down approximately one-sixth the normal reaction volume. The index adapter PCR and Ampure XP bead purification steps were scaled down to approximately two-thirteenths the normal reaction volume. The final library resuspension volume was 29 µl. Of each library, 1 µl was pooled for an initial shallow NGS run on a MiSeq (Illumina) using a Nano flow cell. This equal volume pool was used to estimate the differential volumes required for similar read depths across samples using a NovaSeq SP or S4 flow cell (Illumina). Between 5 µl and 0.2 µl of library material, depending on the data provided from the MiSeq Nano run, was pipetted into a single pool for the NovaSeq run. Transfer volumes were capped at 5 µl to reduce pipetting time and because these types of ‘high-volume’ samples typically contained a higher proportion of likely adapter dimers that inhibit flow cell performance for all samples. A Dragonfly Discovery (SPT Labtech) was used to dispense reaction master mixes or water depending on the step. A BlueWasher (BlueCatBio) was used for high-throughput centrifugal 384-well plate washing during the AmpureXP bead reaction cleanup steps. An IKA MS3 Control linear plate mixer (IKA Works Inc.) set to 2,600 r.p.m. for 5 min was used to resuspend the AmpureXP beads during the rehydration steps. A Mosquito Genomics HV 16 channel robotic liquid handler (SPT Labtech) was used to dispense the RNA, the reaction master mixes and prepare the equal volume pools for the initial MiSeq Nano (Illumina) balancing runs. A Mosquito X1 single-channel ‘hit picker’ robotic liquid handler (SPT Labtech) was used for the final library balancing for the NovaSeq (Illumina) NGS lanes.

Sequencing data were analysed using the C-VIEW (COVID-19 Viral Epidemiology Workflow) platform for initial quality control and SARS-CoV-2 lineage assignment and phylogenetics. In brief, sequencing reads were aligned with minimap2 (ref. ^30^), and primer sequence trimming and quality filtering were applied using the iVar trim method^20^. Sequencing depth and SNV calls were obtained using samtools mpileup^31^ and the iVar variants method^20^.

Controls were included at all stages of sample processing (viral concentration, extraction, qPCR and sequencing) to assess potential inhibition and cross-contamination. Most of the sample processing steps were performed by liquid handling robots for consistency and to minimize human error. Replicates were included for all wastewater samples. If any of the controls failed or indicated cross-contamination, the entire batch was rerun. The clinical samples and wastewater samples were processed separately for sequencing due to significant differences in viral load between the two sample types.

### Virus diversity

As reported previously^20^, virus SNVs were used to characterize the populations derived from wastewater and clinical samples. Richness was defined as the total number of SNV sites, and mean Shannon entropy $H\left(p\right)$ was defined as:

$$H\left(p\right)=\frac{1}{N}\mathop{\sum }\limits_{i=1}^{N}-{p}_{i}{\log }_{2}{p}_{i}-(1-{p}_{i}){\log }_{2}(1-{p}_{i})$$

where ${p}_{i}$ is the SNV frequency of at the *i*-th site, of *N* total sites. For statistical testing, a Mann–Whitney *U*-test was performed using all wastewater samples that were not sampled from the same source within a 10-day period to maximize independence across samples, as well as all clinical samples. Effect size was calculated using the rank-biserial correlation, $r=\frac{2U}{{n}_{{\rm{w}}{\rm{w}}}{n}_{{\rm{c}}{\rm{s}}}}-1$, where *U* is the Mann–Whitney test statistic and ${n}_{{\rm{w}}{\rm{w}}}$ and ${n}_{{\rm{c}}{\rm{s}}}$ are the numbers of wastewater and clinical samples, respectively.

### Wastewater sample deconvolution

To infer relative abundance within a wastewater sample, we used a ‘barcode’ matrix containing the lineage-defining mutations for each known virus lineage:

$$A=\left[\begin{array}{ccc}{a}_{\mathrm{1,1}} & \cdots  & {a}_{1,N}\\ \vdots  & \ddots  & \vdots \\ {a}_{M,N} & \cdots  & {a}_{M,N}\end{array}\right]$$

where ${a}_{i,j}$ denotes the *i*-th lineage at mutation *j*. Lineage-defining mutations were obtained from the UShER global phylogenetic tree using the matUtils package^15^. Similarly, we let *b* and *d* encode the frequency of each mutation and the corresponding sequencing depth (using the log-transform ${d}_{i}={\log }_{2}({{\rm{depth}}}_{i}+1)$ to adjust for large differences in depth across amplicons, which we use to control for heteroskedasticity and down-weight the importance of sites with little or no sequencing depth):

$$b=\left[\begin{array}{c}{b}_{1}\\ \vdots \\ {b}_{N}\end{array}\right],{d}=\left[\begin{array}{c}{d}_{1}\\ \vdots \\ {d}_{N}\end{array}\right]$$

We were then able to write this as a constrained (weighted) least absolute deviations problem:

$$\hat{x}=\,\mathop{{\rm{a}}{\rm{r}}{\rm{g}}{\rm{m}}{\rm{i}}{\rm{n}}}\limits_{\begin{array}{c}x\ge 0\\ \sum x=1\end{array}}\,{\Vert {A}^{{\rm{T}}}x-b\Vert }_{1W},{\rm{w}}{\rm{h}}{\rm{e}}{\rm{r}}{\rm{e}}{\Vert \mu \Vert }_{1W}={\sum }_{i=1}^{N}{d}_{i}|{\mu }_{i}|$$

which yields the ‘demixing’ vector $\hat{x}=[{\hat{x}}_{1}\ldots {\hat{x}}_{M}]$ that specifies the relative abundances of each of the known haplotypes. Analysis was only performed on samples with greater than 70% coverage, with the exception of March samples from the UCSD for which all samples with greater than 50% coverage were used. Constrained minimization was performed in Python using the cvxpy convex optimization package^32,33^. Mapping of lineages to variant WHO lineages (VOCs, VOIs and so on) was performed using curated lineage data from outbreak.info^6^. We note that the Epsilon variant received different maximum escalation levels at the US CDC and WHO, which assigned VOC and VOI status, respectively. As the Epsilon variant was widespread in California and much of the United States, we used the more ‘local’ CDC designation.

### Fast bootstrapping method

Bootstrapping was performed at the nucleotide level by resampling each site based on a multinomial distribution of read depth across all sites, where the event probabilities were determined by the fraction of the total sample reads found at each site, followed by a secondary resampling at each site according to a multinomial distribution (that is, binomial when there was only one SNV at a site), where event probabilities were determined by the frequencies of each base at the site, and the number of trials is given by the sequencing depth. Resamplings and demixings (*n* = 1,000) were performed for all samples.

### Spike-in mixture experiment

RNA was isolated from supernatants of a mammalian cell culture infected with one of five strains of SARS-CoV-2 (A, B.1.1.7, B.1.351, P.1 or B.1.617.2).

#### RNA concentration standardization

Virus concentration was quantified by the UCSD EXCITE COVID testing laboratory using the Thermo COVID-19 Test kit (PN:A47814, Thermo Scientific Corporation). The median cycle quantification (Cq) values (N-gene, Orf1ab and S-gene (where applicable)) were calculated and used to determine how much the RNA needed to be diluted with water to reach a Cq value of 23. A post-dilution RT–qPCR was performed and used to calculate the final dilution of the more concentrated samples to a new target value of Cq of 23.296. The number of freeze–thaw cycles between RNA samples was kept the same.

#### Virus mixing

RNA standardized in the previous section was used to make a volumetric mixing array (final volume of 10 µl) using a Mosquito X1 HV robotic liquid handler (SPT Labtech). Pairwise mixes of 5:95, 10:90, 20:80, 60:40 and 50:50 were made for each virus lineage and in both directions. Equal mixes (20%) for each of the five test strains were made. 25% mixes and 33% mixes were made for a subset of possible combinations and controls of 100:0 were prepared. See Extended Data Table 1 for complete array. Corrected estimates of the fraction of each virus lineage were performed using the final measured Cq values for each pure virus lineage sample to control for issues encountered during the dilution step (repeat Cq measurements had a coefficient of variation of 0.007; Extended Data Table 2). Across all 95 mixtures, we observed a coefficient of variation of 0.016. As initial virus concentrations were controlled for using measured Cq values, we expect that remaining lineage-specific bias (Extended Data Fig. 3) is probably due to experimental inconsistencies encountered during mixture creation.

### Deconvolution method performance comparison

A subset of the spike-in mixtures (one of each type, for a total of 95 mixtures) was used to compare Freyja^34^, cojac (using VOC definitions from the public cojac GitHub repository; lineage A and Epsilon definitions were created manually), the Kallisto-based method from Baaijens et al.^12^ and LCS. Kallisto was run using 10 cores (with no bootstrapping), and LCS was run using 16 cores, both on an Intel Xeon processor (2.2 GHz). LCS was run for 48 h, but failed to complete. Timing was performed using the ‘time’ command, and included all steps after alignment, trimming and sorting. Times correspond to total CPU time.

### Estimation of delay in detection frequency

Estimation of the lag time between epidemiological curves for wastewater and clinical surveillance of the Epsilon variant in San Diego was performed by identifying the shift with maximal cross-correlation. All time points leading up to the time of initial peak in detection frequency were included for both wastewater and clinical data.

### Phylogenetic analyses

Reconstruction of maximum likelihood trees was performed on all SARS-CoV-2 VOC genomes with 10× (10 reads or greater per site) genome coverage of more than 95% and quality score of more than 20 obtained from UCSD campus sampling, using IQtree^35^. This analysis included 150 (112 clinical and 38 wastewater) Epsilon, 49 (37 clinical and 12 wastewater) Alpha, and 160 (136 clinical and 24 wastewater) Delta lineage genomes from the UCSD, in addition to 60 Epsilon, 20 Alpha and 39 Delta randomly selected genomes from elsewhere in the United States. We used iVar^20^ to identify consensus sequences for all San Diego samples. Bases were only included in the sequence if there was a consensus base at the site (more than 50% nucleotide frequency). We also masked known homoplasic sites before tree reconstruction^36^. Analysis of temporal comparison was performed on 608 samples (443 clinical and 165 wastewater, all lineages were included) with 10× genome coverage of more than 95% and quality score of 20 from the UCSD. Sample collection SNP distances were calculated without considering ambiguous bases and gaps.

### Statistics and reproducibility

Experiments for retrieving sequences from samples reported in Fig. 5 and Extended Data Fig. 5C were run twice along with positive (spike-in controls of known SARS-CoV-2 lineages derived from mammalian cells as well as heat-inactivated SARS-CoV-2 viral particles in wastewater) and negative controls. Experiments were repeated twice for a batch of 207 wastewater samples. All attempts at replication were successful. For spike-in data reported in Fig. 2 and Extended Data Fig. 3, extraction and RT–qPCR for spike-ins of lineage A from clinical samples were repeated with 20 replicates to check for overall assay variability (reported in Extended Data Table 2).

### Reporting summary

Further information on research design is available in the Nature Research Reporting Summary linked to this article.

## Online content

Any methods, additional references, Nature Research reporting summaries, source data, extended data, supplementary information, acknowledgements, peer review information; details of author contributions and competing interests; and statements of data and code availability are available at 10.1038/s41586-022-05049-6.

### Supplementary information

Reporting Summary

## Figures and Tables

**Extended Data Table 1:**
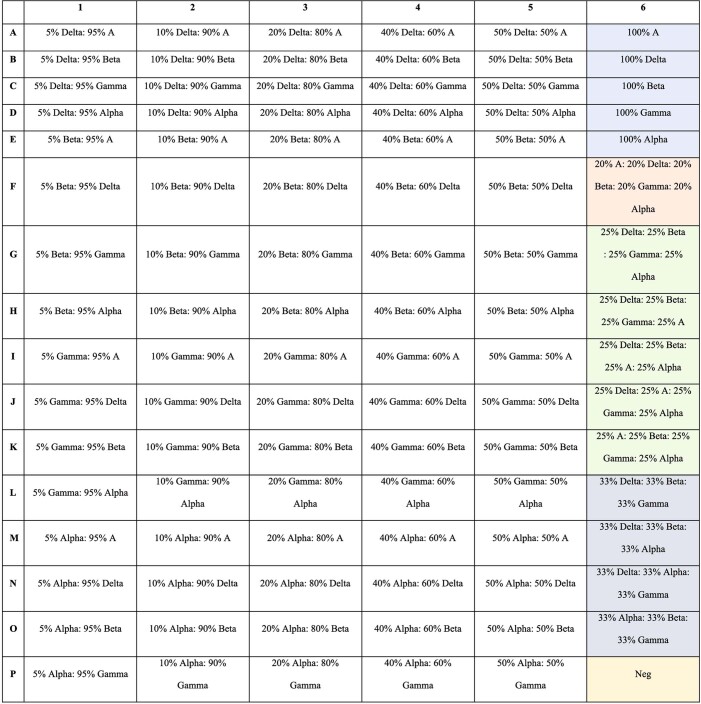
Plate map of spike-in mixtures used for method validation

**Extended Data Table 2:**
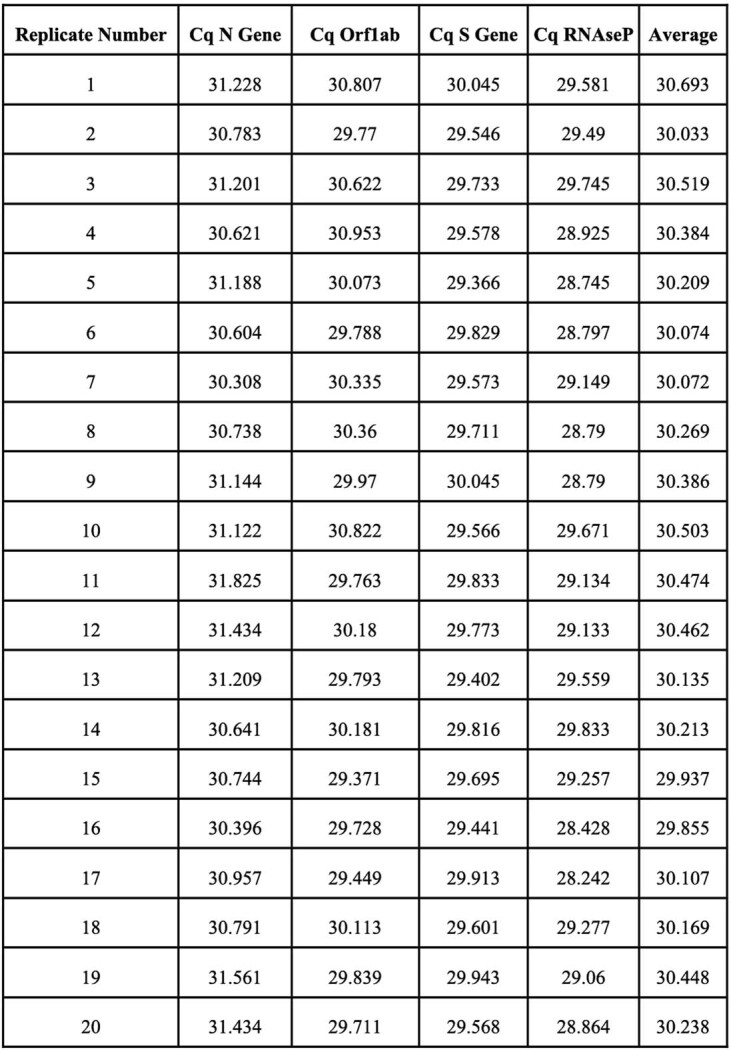
Consistency of Lineage A Cq values across repeated measurements

**Extended Data Table 3:**
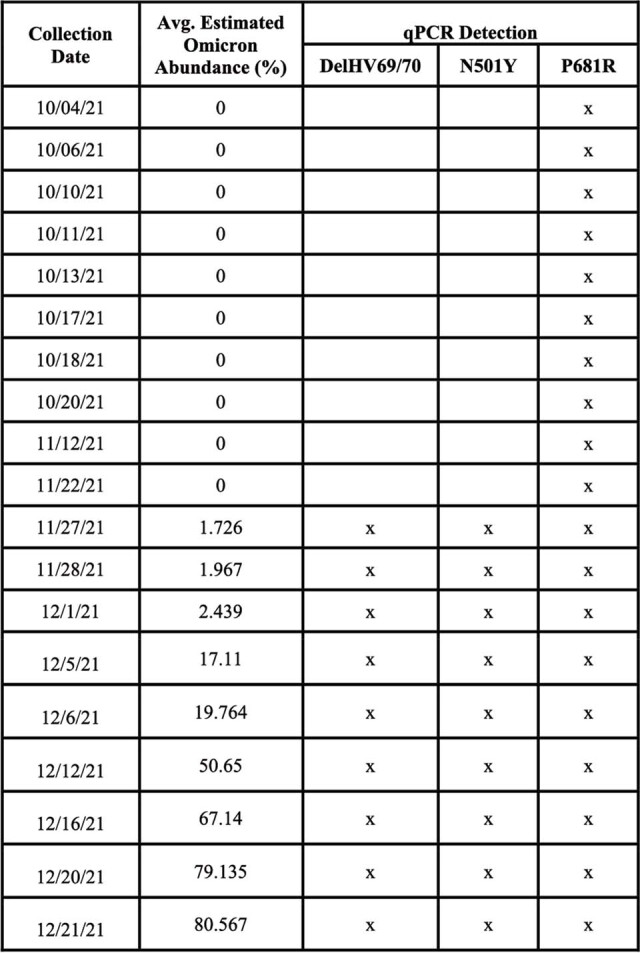
Omicron surveillance at Point Loma Wastewater Treatment Plant

## Data Availability

All raw wastewater sequencing data are available via the NCBI Sequence Read Archive under the BioProject ID PRJNA819090. Consensus sequences from clinical and wastewater surveillance are all available on GISAID. Spike-in sequencing data are available via Google cloud (https://console.cloud.google.com/storage/browser/search-reference_data). The UCSD campus dashboard can be accessed at https://returntolearn.ucsd.edu/dashboard/. The county wastewater data from Point Loma are available through the public dashboard at https://searchcovid.info/dashboards/wastewater-surveillance/. The SEARCH genomic surveillance dashboard is available at https://searchcovid.info/dashboards/sequencing-statistics/.

## Code availability

Freyja is hosted publicly on GitHub (https://github.com/andersen-lab/Freyja) and is available under a BSD-2-Clause license (10.5281/zenodo.6585067, all versions). Freyja is accessible as a package via bioconda (https://bioconda.github.io/recipes/freyja/README.html) in container form via dockerhub (https://hub.docker.com/r/andersenlabapps/freyja). COVID-19 Viral Epidemiology Workflow (C-VIEW) is available at https://github.com/ucsd-ccbb/C-VIEW as an open-source, end-to-end workflow for viral epidemiology focused on SARS-CoV-2 lineage assignment and phylogenetics. C-VIEW uses minimap2 (v2.17), samtools(v1.11), iVar(v1.3.1) and pangolin (varying versions).

## References

[1] Reitsma, M. B. et al. Racial/ethnic disparities in COVID-19 exposure risk, testing, and cases at the subcounty level in California. *Health Aff.* **40**, 870–878 (2021). 10.1377/hlthaff.2021.00098 PMC8458028 33979192

[2] Lieberman-Cribbin, W., Tuminello, S., Flores, R. M. & Taioli, E. Disparities in COVID-19 testing and positivity in New York City. *Am. J. Prev. Med.* **59**, 326–332 (2020). 32703702 10.1016/j.amepre.2020.06.005 PMC7316038

[3] Brito, A. F. et al. Global disparities in SARS-CoV-2 genomic surveillance. Preprint at *medRxiv*10.1101/2021.08.21.21262393 (2021).

[4] Karthikeyan, S. et al. High-throughput wastewater SARS-CoV-2 detection enables forecasting of community infection dynamics in San Diego County. *mSystems* **6**, e00045-21 (2021). 33653938 10.1128/mSystems.00045-21 PMC8546963

[5] Randazzo, W. et al. SARS-CoV-2 RNA in wastewater anticipated COVID-19 occurrence in a low prevalence area. *Water Res.* **181**, 115942 (2020). 32425251 10.1016/j.watres.2020.115942 PMC7229723

[6] Mullen, J. L., the Center for Viral Systems Biology et al. *outbreak.info* https://outbreak.info/ (2021).

[7] Harvey, W. T. et al. SARS-CoV-2 variants, spike mutations and immune escape. *Nat. Rev. Microbiol.* **19**, 409–424 (2021). 34075212 10.1038/s41579-021-00573-0 PMC8167834

[8] Hata, A., Hara-Yamamura, H., Meuchi, Y., Imai, S. & Honda, R. Detection of SARS-CoV-2 in wastewater in Japan during a COVID-19 outbreak. *Sci. Total Environ.* **758**, 143578 (2021). 33221007 10.1016/j.scitotenv.2020.143578 PMC7654358

[9] Karthikeyan, S. et al. Rapid, large-scale wastewater surveillance and automated reporting system enable early detection of nearly 85% of COVID-19 cases on a university campus. *mSystems* **6**, e0079321 (2021). 34374562 10.1128/mSystems.00793-21 PMC8409724

[10] Mercer, T. R. & Salit, M. Testing at scale during the COVID-19 pandemic. *Nat. Rev. Genet.* **22**, 415–426 (2021). 33948037 10.1038/s41576-021-00360-w PMC8094986

[11] Crits-Christoph, A. et al. Genome sequencing of sewage detects regionally prevalent SARS-CoV-2 variants. *mBio* **12**, e02703-20 (2021). 33468686 10.1128/mBio.02703-20 PMC7845645

[12] Baaijens, J. A. et al. Variant abundance estimation for SARS-CoV-2 in wastewater using RNA-seq quantification. Preprint at *medRxiv*10.1101/2021.08.31.21262938 (2021).

[13] Amman, F. et al. Viral variant-resolved wastewater surveillance of SARS-CoV-2 at national scale. *Nat. Biotechnol.*10.1038/s41587-022-01387-y (2022). 10.1038/s41587-022-01387-y 35851376

[14] Rambaut, A. et al. A dynamic nomenclature proposal for SARS-CoV-2 lineages to assist genomic epidemiology. *Nat. Microbiol.* **5**, 1403–1407 (2020). 32669681 10.1038/s41564-020-0770-5 PMC7610519

[15] Turakhia, Y. et al. Ultrafast sample placement on existing tRees (UShER) enables real-time phylogenetics for the SARS-CoV-2 pandemic. *Nat. Genet.* **53**, 809–816 (2021). 33972780 10.1038/s41588-021-00862-7 PMC9248294

[16] Walke, H. T., Honein, M. A. & Redfield, R. R. Preventing and responding to COVID-19 on college campuses. *JAMA* **324**, 1727–1728 (2020). 32991681 10.1001/jama.2020.20027 PMC9648565

[17] Fielding-Miller, R. K. et al. Wastewater and surface monitoring to detect COVID-19 in elementary school settings: the Safer at School Early Alert project. Preprint at *medRxiv*10.1101/2021.10.19.21265226 (2021).

[18] Ladner, J. T., Grubaugh, N. D., Pybus, O. G. & Andersen, K. G. Precision epidemiology for infectious disease control. *Nat. Med.* **25**, 206–211 (2019). 30728537 10.1038/s41591-019-0345-2 PMC7095960

[19] Grubaugh, N. D. et al. Tracking virus outbreaks in the twenty-first century. *Nat. Microbiol.* **4**, 10–19 (2019). 30546099 10.1038/s41564-018-0296-2 PMC6345516

[20] Grubaugh, N. D. et al. An amplicon-based sequencing framework for accurately measuring intrahost virus diversity using PrimalSeq and iVar. *Genome Biol.* **20**, 8 (2019). 30621750 10.1186/s13059-018-1618-7 PMC6325816

[21] McBroome, J. et al. A daily-updated database and tools for comprehensive SARS-CoV-2 mutation-annotated trees. *Mol. Biol. Evol.* **38**, 5819–5824 (2021). 34469548 10.1093/molbev/msab264 PMC8662617

[22] Jahn, K. et al. Early detection and surveillance of SARS-CoV-2 genomic variants in wastewater using COJAC. *Nat. Microbiol.* **7**, 1151–1160 (2022). 10.1038/s41564-022-01185-x PMC9352586 35851854

[23] Valieris, R. et al. A mixture model for determining SARS-Cov-2 variant composition in pooled samples. *Bioinformatics* **38**, 1809–1815 (2022). 35104309 10.1093/bioinformatics/btac047

[24] Peccia, J. et al. Measurement of SARS-CoV-2 RNA in wastewater tracks community infection dynamics. *Nat. Biotechnol.* **38**, 1164–1167 (2020). 32948856 10.1038/s41587-020-0684-z PMC8325066

[25] Singanayagam, A. et al. Community transmission and viral load kinetics of the SARS-CoV-2 Delta (B.1.617.2) variant in vaccinated and unvaccinated individuals in the UK: a prospective, longitudinal, cohort study. *Lancet Infect. Dis.* **22**, 183–195 (2022). 34756186 10.1016/S1473-3099(21)00648-4 PMC8554486

[26] Cevik, M. et al. SARS-CoV-2, SARS-CoV, and MERS-CoV viral load dynamics, duration of viral shedding, and infectiousness: a systematic review and meta-analysis. *Lancet Microbe* **2**, e13–e22 (2021). 33521734 10.1016/S2666-5247(20)30172-5 PMC7837230

[27] Wu, Y. et al. Prolonged presence of SARS-CoV-2 viral RNA in faecal samples. *Lancet Gastroenterol. Hepatol.* **5**, 434–435 (2020). 32199469 10.1016/S2468-1253(20)30083-2 PMC7158584

[28] Xu, Y. et al. Characteristics of pediatric SARS-CoV-2 infection and potential evidence for persistent fecal viral shedding. *Nat. Med.* **26**, 502–505 (2020). 32284613 10.1038/s41591-020-0817-4 PMC7095102

[29] Goyal, R., Hotchkiss, J., Schooley, R. T., De Gruttola, V. & Martin, N. K. Evaluation of SARS-CoV-2 transmission mitigation strategies on a university campus using an agent-based network model. *Clin. Infect. Dis.* **73**, 1735–1741 (2021). 33462589 10.1093/cid/ciab037 PMC7929036

[30] Li, H. Minimap2: pairwise alignment for nucleotide sequences. *Bioinformatics* **34**, 3094–3100 (2018). 29750242 10.1093/bioinformatics/bty191 PMC6137996

[31] Li, H. et al. The Sequence Alignment/Map format and SAMtools. *Bioinformatics* **25**, 2078–2079 (2009). 19505943 10.1093/bioinformatics/btp352 PMC2723002

[32] Diamond, S. & Boyd, S. CVXPY: a Python-embedded modeling language for convex optimization. *J. Mach. Learn. Res.* **17**, 83 (2016). 27375369 PMC4927437

[33] Agrawal, A., Verschueren, R., Diamond, S. & Boyd, S. A rewriting system for convex optimization problems. *J. Control Decision* **5**, 42–60 (2018). 10.1080/23307706.2017.1397554

[34] Levy, J. I., McDonald, D., Tomkins-Tinch, C. & Petit, R. A. III. andersen-lab/Freyja: 1.3.7. Zenodo 10.5281/zenodo.6585068 (2022).

[35] Minh, B. Q. et al. IQ-TREE 2: new models and efficient methods for phylogenetic inference in the genomic era. *Mol. Biol. Evol.* **37**, 1530–1534 (2020). 32011700 10.1093/molbev/msaa015 PMC7182206

[36] Issues with SARS-CoV-2 sequencing data. *Virological* https://virological.org/t/issues-with-sars-cov-2-sequencing-data/473 (2020).

