# Supplementary material for: Wastewater sequencing reveals early cryptic SARS-CoV-2 variant transmission
Source: Nature. 2022 Jul 7;609(7925):101–8. doi: 10.1038/s41586-022-05049-6 (PMC9433318; doi:10.1038/s41586-022-05049-6)
Supplement: Supplementary file 1 — Reporting Summary [file 41586_2022_5049_MOESM1_ESM.pdf]

nature portfolio

Corresponding author(s): Rob Knight

Last updated by author(s): May 30, 2022

Reporting Summary

Nature Portfolio wishes to improve the reproducibility of the work that we publish. This form provides structure for consistency and transparency in reporting. For further information on Nature Portfolio policies, see our Editorial Policies and the Editorial Policy Checklist.

Statistics

For all statistical analyses, confirm that the following items are present in the figure legend, table legend, main text, or Methods section.

n/a	Confirmed	
☐	☒	The exact sample size (*n*) for each experimental group/condition, given as a discrete number and unit of measurement
☒	☐	A statement on whether measurements were taken from distinct samples or whether the same sample was measured repeatedly
☐	☒	The statistical test(s) used AND whether they are one- or two-sided<br>*Only common tests should be described solely by name; describe more complex techniques in the Methods section.*
☒	☐	A description of all covariates tested
☒	☐	A description of any assumptions or corrections, such as tests of normality and adjustment for multiple comparisons
☐	☒	A full description of the statistical parameters including central tendency (e.g. means) or other basic estimates (e.g. regression coefficient) AND variation (e.g. standard deviation) or associated estimates of uncertainty (e.g. confidence intervals)
☐	☒	For null hypothesis testing, the test statistic (e.g. *F*, *t*, *r*) with confidence intervals, effect sizes, degrees of freedom and *P* value noted<br>*Give P values as exact values whenever suitable.*
☒	☐	For Bayesian analysis, information on the choice of priors and Markov chain Monte Carlo settings
☒	☐	For hierarchical and complex designs, identification of the appropriate level for tests and full reporting of outcomes
☒	☐	Estimates of effect sizes (e.g. Cohen's *d*, Pearson's *r*), indicating how they were calculated

Our web collection on statistics for biologists contains articles on many of the points above.

Software and code

Policy information about availability of computer code

Data collection	All data collection code is publicly available in our COVID-19 Viral Epidemiology Workflow (C-VIEW) at https://github.com/ucsd-ccbb/C-VIEW as an open-source, end-to-end workflow for viral epidemiology focused on SARS-CoV-2 lineage assignment and phylogenetics. C-VIEW uses minimap2 (v2.17), samtools(v1.11), iVar(v1.3.1), and pangolin (varying versions).
Data analysis	All data analysis is performed using Freyja (v.1.3.7) as well as custom Python 3 scripts. Freyja is hosted publicly on github (https://github.com/andersen-lab/Freyja) and is available under a BSD-2-Clause License (doi: 10.5281/zenodo.6585067, version 1.3.7). Freyja is accessible as a package via bioconda (https://bioconda.github.io/recipes/freyja/README.html) in container form via dockerhub (https://hub.docker.com/r/andersenlabapps/freyja).

For manuscripts utilizing custom algorithms or software that are central to the research but not yet described in published literature, software must be made available to editors and reviewers. We strongly encourage code deposition in a community repository (e.g. GitHub). See the Nature Portfolio guidelines for submitting code & software for further information.

Data

Policy information about availability of data

All manuscripts must include a data availability statement. This statement should provide the following information, where applicable:

- Accession codes, unique identifiers, or web links for publicly available datasets
- A description of any restrictions on data availability
- For clinical datasets or third party data, please ensure that the statement adheres to our policy

All raw wastewater sequencing data is available via the NCBI Sequence Read Archive under the BioProject ID PRJNA819090. Spike-in sequencing data is available via google cloud (https://console.cloud.google.com/storage/browser/search-reference_data). The UCSD campus dashboard can be accessed at https://returntolearn.ucsd.edu/dashboard/ . The county wastewater data from Point Loma are available through the public dashboard that can be accessed at https://searchcovid.info/dashboards/wastewater-surveillance/. The SEARCH genomic surveillance dashboard is available at https://searchcovid.info/dashboards/sequencing-statistics/. Consensus sequences from clinical and wastewater surveillance are all available on GISAID. Further details are provided here: https://github.com/andersen-lab/ HCoV-19-Genomics.

Human research participants

Policy information about studies involving human research participants and Sex and Gender in Research.

Reporting on sex and gender	Sex/gender-based data were not considered in this study design.
Population characteristics	No covariate relevant population characteristics (age, gender etc.) was used in this study
Recruitment	Participants were recruited on the basis that they had a positive COVID-19 test at UCSD, as approved under a waiver of consent framework, or on the basis that they volunteered for an associated study. Both of these pathways for recruitment were IRB-approved (see below).
Ethics oversight	The University of California San Diego Institutional Review Boards (IRB) provided human subject protection oversight of the of the data obtained by the EXCITE CLIA lab for the campus clinical samples (IRB approval #210699, #200477). All necessary patient/participant consent has been obtained and the appropriate institutional forms have been archived, and any sample identifiers included were de-identified. The wastewater component of this project was discussed with our Institutional Review Board, and was not deemed to be human subject research as it did not record personally identifiable information.

Note that full information on the approval of the study protocol must also be provided in the manuscript.

Field-specific reporting

Please select the one below that is the best fit for your research. If you are not sure, read the appropriate sections before making your selection.

☐ Life sciences ☐ Behavioural & social sciences ☒ Ecological, evolutionary & environmental sciences

For a reference copy of the document with all sections, see nature.com/documents/nr-reporting-summary-flat.pdf

Ecological, evolutionary & environmental sciences study design

All studies must disclose on these points even when the disclosure is negative.

Study description	To compare the relative utility of wastewater genomic surveillance in enabling early detection of SARS-CoV-2 VOIs/VOCs and transmission dynamics, a high-resolution 295-day wastewater and clinical sequencing effort was performed in the controlled environment of a large university campus and the broader context of the surrounding county.
Research sample	Between November 2020-September 2021, 21,383 wastewater samples were collected from UCSD campus and the surrounding county and analyzed for presence of SARS-CoV-2 viral RNA. The campus wastewater samples covered 343 campus buildings representing a catchment of approximately 25,000 students and staff (capturing all on-campus residents). The wastewater treatment plant at Point Loma covers a catchment of 2.2 million residents in the greater San Diego county. The samples from the public school districts cover a combined population of 10,000 students and staff. Samples positive for the RNA as determined via qPCR were sequenced using a miniaturized tiled amplicon approach. During the same period of study, clinical samples were also sequenced from the UCSD campus as well the surrounding county. Sequencing of 600 campus wastewater samples were compared to 759 genomes obtained from campus clinical swabs (processed by the CALM and EXCITE CLIA labs at UCSD). 31,149 genomes obtained from clinical genomic surveillance of the greater San Diego community were compared to 837 wastewater sample sequences collected from San Diego county.
Sampling strategy	Sampling sites were identified via campus GIS, which provides mapping and flow direction of interconnected sewer lines and locations of manholes where samplers could be placed. In the UCSD campus, wastewater autosamplers were first prioritized to the most proximal manhole to buildings with residential populations greater than 150. This decision was made based on agent-based

	network modeling of SARS-CoV-2 transmission on the UCSD campus, indicating that the highest risk areas for large outbreaks on campus were buildings containing the largest residential populations (details provided in Goyal et al 2021, see References). 131 wastewater autosamplers were placed optimally to cover 343 campus buildings thereby capturing the majority of the campus population. For the San Diego county samples, the main wastewater treatment plant was chosen as the sampling site since it captured a majority (2.2 million) of the area's residents. For the public school districts, pilot sites were selected from ZIP Codes with COVID-19 rates above the county median and with high levels of social vulnerability according to the California Healthy Places Index. The samplers for the campus and school districts were placed at manholes or sewer cleanouts such that they captured the waste from all the schools/building's residents.<br>Sampling procedures are described in detail at: dx.doi.org/10.17504/protocols.io.bshvnb66.
Data collection	Sample data logging was streamlined via integration with the campus GIS (geographic information system) server. The spatially enabled sewer network and subsequent trace of samplers to buildings were stored in and performed by ArcGIS Pro 2.7 (Esri). Details on sample collection and data integration are also provided at: https://doi.org/l0.1128/mSystems.00793-21. The unique autosampler barcode and the sample bottle barcodes were scanned by the sample collection staff using the ArcGIS Survey123 mobile app (ESRI) which enabled automatic data integration into the ArcGIS Online environment for trace analysis.
Timing and spatial scale	During the 10 month study period, wastewater samples were collected on a daily basis from the UCSD campus from 131 sampling sites covering nearly 350 campus buildings. Wastewater samples were collected 5 days a week from the public schools in the various San Diego school districts and 3 times a week from the Point Loma wastewater treatment plant (the primary plant serving the greater San Diego area). This is part of an ongoing study and samples continue to be collected from these sites regularly as of May 2022. The spatial scale of the campus data is shown in Fig. 1A of the current manuscript. The spatial scale of the county samples are provided in Karthikeyan et al., 2021 (doi: 10.1128/mSystems.00045-21) and Fielding-Miller et al., 2021 (doi: /10.1101/2021.10.19.21265226)
Data exclusions	All wastewater sequences used had greater than 70% coverage, with the exception of March samples from UCSD for which all samples with greater than 50% coverage were used due to low sample numbers during that period. No data were excluded from the study unless they failed to meet the quality threshold specified above (70% coverage of the SARS-CoV-2 genome with no evidence of cross-contamination as well as the positive and negative controls passing QC for the specific run). 4.7% of the sequenced samples failed to meet the QC threshold.
Reproducibility	Controls were included at all stages of sample processing (viral concentration, extraction, qPCR and sequencing) to assess potential inhibition and cross contamination. Most of the sample processing steps were performed by liquid handling robots to minimize human error and replicates included. If any of the controls failed or indicated cross-contamination, the entire batch was rerun. The clinical samples and wastewater samples were processed separately for sequencing due to significant differences in viral load between the two sample types. Due to the sample heterogeneity in complex environmental matrices such as wastewater, controls are vital in aiding of data interpretation. With every sequencing run spike-in controls of a known SARS-CoV-2 lineage (Lineage A) was chosen as a positive control and a no template extraction blank was used as a negative control to assess cross-contamination during the library generation and the sequencing stage. If significant SARS-CoV-2 mapping reads were found passing QC in the negative controls, the entire batch was rerun from the library generation step. Experiments for retrieving sequences from samples reported in Fig. 5 and Extended Data Figure 5C were run twice along with positive (spike-in controls of known SARS-CoV-2 lineages derived from mammalian cells as well as heat-inactivated SARS-CoV-2 viral particles in wastewater) and negative controls. Experiments were repeated twice for a batch of 207 wastewater samples. All attempts at replication were successful. For spike-in data reported in Fig 2 and Extended Data Fig. 3, extraction and RT-qPCR for spike-ins of Lineage A from clinical samples were repeated with 20 replicates to check for overall assay variability (reported in Extended Data Table 2).<br>Detailed wastewater sample processing steps are also provided here: dx.doi.org/10.17504/protocols.io.bshvnb66 as well as in the Methods section of the manuscript
Randomization	All sequences/samples that met our threshold for quality were used in the study. Randomization was not required since we did not perform experiments to evaluate the effects of specific treatments or groups.
Blinding	Blinding was not applicable to this study as we did not perform experiments involving specific treatments or groups and all clinical data used were de-identified from the point of receipt.

Did the study involve field work? ☐ Yes ☒ No

Reporting for specific materials, systems and methods

We require information from authors about some types of materials, experimental systems and methods used in many studies. Here, indicate whether each material, system or method listed is relevant to your study. If you are not sure if a list item applies to your research, read the appropriate section before selecting a response.

Materials & experimental systems

n/a	Involved in the study
☒	☐ Antibodies
☒	☐ Eukaryotic cell lines
☒	☐ Palaeontology and archaeology
☒	☐ Animals and other organisms
☒	☐ Clinical data
☒	☐ Dual use research of concern

Methods

n/a	Involved in the study
☒	☐ ChIP-seq
☒	☐ Flow cytometry
☒	☐ MRI-based neuroimaging
